# The Molecular Brakes of Adipose Tissue Lipolysis

**DOI:** 10.3389/fphys.2022.826314

**Published:** 2022-02-24

**Authors:** Yongguo Li, Zhen Li, Devi Anggraini Ngandiri, Mireia Llerins Perez, Alexander Wolf, Yuanyuan Wang

**Affiliations:** Emmy Noether Group for Molecular and Cellular Metabolism, Chair for Molecular Nutritional Medicine, TUM School of Life Sciences, Technical University of Munich, Freising, Germany

**Keywords:** adipocytes, lipolysis, free fatty acid, thermogenesis, molecular brakes, feedback mechanisms, reesterification, lipophagy

## Abstract

Adaptation to changes in energy availability is pivotal for the survival of animals. Adipose tissue, the body’s largest reservoir of energy and a major source of metabolic fuel, exerts a buffering function for fluctuations in nutrient availability. This functional plasticity ranges from energy storage in the form of triglycerides during periods of excess energy intake to energy mobilization *via* lipolysis in the form of free fatty acids for other organs during states of energy demands. The subtle balance between energy storage and mobilization is important for whole-body energy homeostasis; its disruption has been implicated as contributing to the development of insulin resistance, type 2 diabetes and cancer cachexia. As a result, adipocyte lipolysis is tightly regulated by complex regulatory mechanisms involving lipases and hormonal and biochemical signals that have opposing effects. In thermogenic brown and brite adipocytes, lipolysis stimulation is the canonical way for the activation of non-shivering thermogenesis. Lipolysis proceeds in an orderly and delicately regulated manner, with stimulation through cell-surface receptors *via* neurotransmitters, hormones, and autocrine/paracrine factors that activate various intracellular signal transduction pathways and increase kinase activity. The subsequent phosphorylation of perilipins, lipases, and cofactors initiates the translocation of key lipases from the cytoplasm to lipid droplets and enables protein-protein interactions to assemble the lipolytic machinery on the scaffolding perilipins at the surface of lipid droplets. Although activation of lipolysis has been well studied, the feedback fine-tuning is less well appreciated. This review focuses on the molecular brakes of lipolysis and discusses some of the divergent fine-tuning strategies in the negative feedback regulation of lipolysis, including delicate negative feedback loops, intermediary lipid metabolites-mediated allosteric regulation and dynamic protein–protein interactions. As aberrant adipocyte lipolysis is involved in various metabolic diseases and releasing the brakes on lipolysis in thermogenic adipocytes may activate thermogenesis, targeting adipocyte lipolysis is thus of therapeutic interest.

## Introduction

Maintaining whole-body energy homeostasis during times of dramatic fluctuations in energy supply and demand is a fundamental property of animal life. Coping with these energy fluctuations has entrained animals with the ability to orchestrate energy metabolism for optimal substrate storage and use during states of either food surplus or famine, and periods of either rest or increased energy demand (Smith et al., 2018). Deficiency in this ability is implicated in the pathogenesis of metabolic diseases such as insulin resistance (IR), type 2 diabetes (T2D) and cancer cachexia. Adipose tissue is a central metabolic organ that responds to fluctuations in nutrient availability to maintain systemic metabolic homeostasis. The ability of adipocyte to efficiently store energy after meals and release it when needed to other cells in the body is critical for integrating metabolism (Frühbeck et al., 2001). Tightly regulated fat accumulation and mobilization are a central characteristic of energy homeostasis.

In mammals, adipose tissue can be divided into white adipose tissue (WAT), brown adipose tissue (BAT) and beige/brite (brown in white) adipose tissue (Li et al., 2014b; Peirce et al., 2014). While white adipocytes, characterized by a single, large intra-cellular lipid droplet, harbored eccentric nucleus and few mitochondria, store energy in the form of triglycerides (TGs), brown and brite adipocytes, which express unique thermogenic uncoupling protein 1 (UCP1), contain multiple small lipid droplets of varied sizes as well as an almost centrally localized nucleus, and most importantly, equipped with an abundant number of mitochondria, specialize in energy dissipation. Under conditions of positive energy balance, expansion of adipose depots can be driven either by the increase in adipocyte size (hypertrophy) or by the formation of new adipocytes from tissue-resident progenitor differentiation in the process of adipogenesis (hyperplasia), thus protecting peripheral tissues from ectopic lipid deposition (Ghaben and Scherer, 2019). Conversely, during energy-demanding conditions such as exercise, fasting or cold stimulus, these stored fats are mobilized to supply the body with free fatty acids (FFAs) as energy substrates. In thermogenic brown and brite adipose tissues, FFAs activate and fuel the UCP1-dependent non-shivering thermogenesis (Cannon and Nedergaard, 2004; Li et al., 2014a). The thermogenic, energy dissipating phenotype of brown and brite adipocytes gains particular therapeutic interest considering the continuously increasing world-wide burden of obesity.

Mobilization of FFAs from stored TGs in adipocytes requires ester hydrolysis by a sequential enzymatic process called lipolysis, which is a pivotal metabolic reaction for energy mobilization. Once released, FFAs have a variety of fates including oxidation for energy in the form of ATP (adenosine triphosphate), re-esterification back into TGs, and also function as signaling molecules (Papackova and Cahova, 2015; Li et al., 2017). Despite their essential roles, high concentrations of FFAs are toxic because of their limited solubility, amphipathic nature and their active transformation into highly bioactive and cytotoxic lipid species. These destructive effects of FFAs — collectively referred to as “lipotoxicity” — can lead to cellular dysfunction and cell death (Unger, 2002). As such, elevated circulating FA (fatty acid) levels contribute to insulin resistance in both animals and humans (Savage et al., 2007). The findings of increased fat mobilization as well as brown fat thermogenesis during cancer cachexia indicate that dysfunction of adipose tissue contributes to the imbalance of energy homeostasis involved in catabolic wasting (Tsoli et al., 2012). Together with anorexia, the aberrant fat mobilization to generate heat represents a maladaptive response experienced by cancer patients (Tsoli et al., 2016). Therefore, intracellular lipolysis is a critical metabolic process of virtually all eukaryotic cells, and the tight control is important to metabolic health. Lipolysis is thus under tight positive and negative physiological regulation. The disruption of this subtle balance between energy storage and mobilization will result into IR, T2D and cancer cachexia (Kahn et al., 2006; Yang and Mottillo, 2020).

As aberrant adipocyte lipolysis is involved in various metabolic diseases and releasing the brakes on lipolysis in thermogenic adipocytes may activate thermogenesis, targeting adipocyte lipolysis is thus of therapeutic interest. Despite a very detailed knowledge of the biochemical and physiological control of lipolysis activation, the molecular brakes are poorly understood. This review focuses on the molecular brakes of lipolysis and discusses some of the divergent fine-tuning strategies in the negative feedback regulation of lipolysis, including delicate negative feedback loops, intermediary lipid metabolites-mediated allosteric regulation and dynamic protein–protein interactions.

## The Canonical Pathway of Adipocyte Lipolysis

**FIGURE 1:**
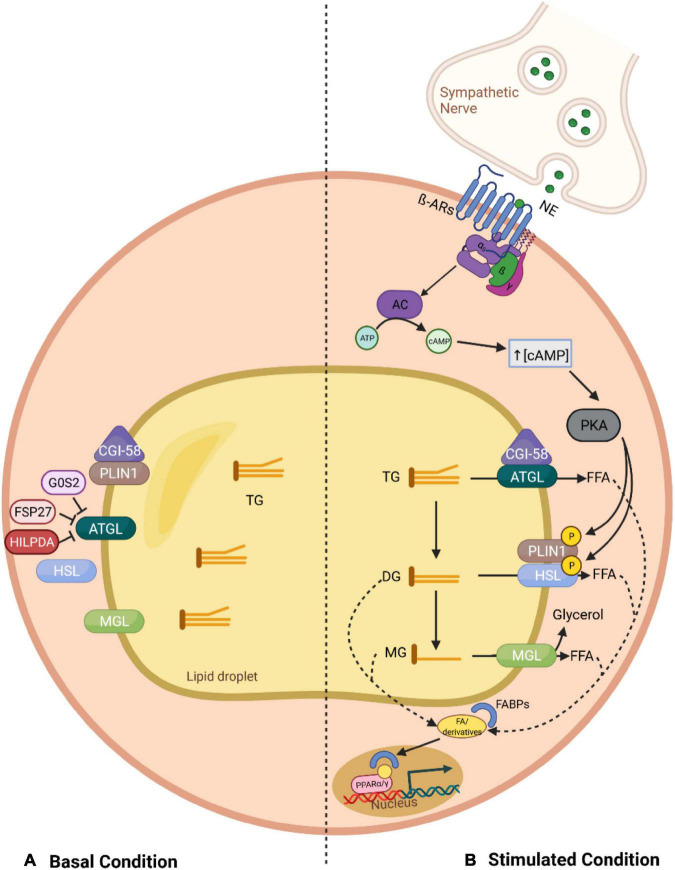
Overview of the canonical lipolysis pathway in adipocytes. **(A)** In the basal status, PLIN1 sequesters CGI-58, the co-activator of ATGL, limiting its function. This, together with the fact that ATGL can be further inhibited through direct interaction with G0S2 and FSP27, leads to a low TG hydrolysis rate. **(B)** In stimulated lipolysis, norepinephrine, secreted by sympathetic nerves innervating the adipose tissue, binds to β adrenergic receptors and activates AC. Consequently, there is an increase in the cytosolic levels of cAMP, which stimulates PKA, a kinase that phosphorylates PLIN1 and HSL. The phosphorylation of PLIN1 results in the release of CGI-58, which can then bind to ATGL, promoting the hydrolysis of TGs into DGs. In addition, upon phosphorylation of HSL, this lipase relocates to the lipid droplet surface where it can interact with PLIN1 and degrades DGs into MGs, which are further processed by MGL. Finally, the fatty acids generated in each lipolytic step, and their derivatives, can act as signaling ligands or as precursors for ligands of PPARα/γ and stimulate the expression of genes involved in energy homeostasis. AC, adenylyl cyclase; AR, adrenergic receptor; ATGL, adipose triglyceride lipase; ATP, adenosine triphosphate; cAMP, cyclic adenosine monophosphate; CGI-58, comparative gene identification 58; DG, diglyceride; FFA, free fatty acid; FABP, fatty acid-binding protein; FSP27, fat specific protein 27; G0S2, G0/S2 switch protein 2; HILPDA, hypoxia-induced lipid droplet-associated protein; HSL, hormone-sensitive lipase; MG, monoglyceride; MGL, monoglyceride lipase; NE, norepinephrine; PKA, protein kinase A; PLIN1, perilipin 1; PPAR, peroxisome proliferator-activated receptor; TG, triglyceride.

In adipocytes, three major neutral lipases participate in the canonical pathway of lipolysis (Figure 1): adipose triglyceride lipase (ATGL)—also termed patatin-like phospholipase domain-containing protein 2 (PNPLA2) or desnutrin, hormone-sensitive lipase (HSL), and monoglyceride lipase (MGL) (Haemmerle et al., 2002; Zimmermann et al., 2004; Taschler et al., 2011). These enzymes operate in a sequential and controlled manner in conjunction with several cofactors to break down TGs stored in lipid droplets. ATGL, considered the rate-limiting enzyme, catalyzes the first step, hydrolyzing TGs to diglycerides (DGs), which in turn are degraded by HSL into monoglycerides, with the release of a FFA in each step (Haemmerle et al., 2002; Zimmermann et al., 2004). Finally, MGL completes the process through the hydrolyzation of the last ester bond in monoglycerides, generating one last FFA and a glycerol backbone (Taschler et al., 2011).

Adipose tissue lipolysis is tightly regulated by endocrine, paracrine, autocrine and autonomic nervous systems, which modulate the activity of lipases by determining their intracellular locations and interactions with regulatory factors. Under non-stimulated conditions (basal), such as feeding, ATGL can be found on the surface of lipid droplets as well as in the cytoplasm, while HSL predominantly remains in the latter (Bezaire et al., 2009). Meanwhile, the lipid droplet-associated protein Perilipin 1 (PLIN1) limits ATGL function by sequestering its co-activator, comparative gene identification 58 (CGI58), also known as ABHD5 (α-β Hydrolase domain-containing protein 5) (Girousse and Langin, 2012). Other accessory proteins can further inhibit ATGL activity through their direct protein-protein interaction with ATGL, including G0/S2 switch protein 2 (G0S2), hypoxia-induced lipid droplet-associated protein (HILPDA), and fat specific protein 27 (FSP27) (Yang X. et al., 2010; Grahn et al., 2014). As a result, the rate of TG hydrolysis within the basal state is low (Figure 1).

In contrast, during periods of high energy demand, such as fasting or cold exposure, lipolysis is activated mainly *via* the action of catecholamines (Heeren and Scheja, 2018). Upon stimulation, sympathetic nerve fibers innervating the adipose tissue release norepinephrine, which binds to β-adrenergic receptors (β-ARs) located on the plasma membrane of adipocytes. Hormonal binding initiates a stimulatory G protein (Gs)-mediated cascade, elevating intracellular levels of the second messenger cyclic AMP (cAMP, cyclic adenosine monophosphate), through the activity of adenylyl cyclase (AC). An accumulation of cAMP in the cytoplasm promotes protein kinase A (PKA) activation, which in turn modulates lipolysis *via* phosphorylation of key effector factors (Langin, 2006). For instance, the phosphorylation of PLIN1 leads to the release of CGI58, enabling it to interact with ATGL, therefore, fully activating its TG hydrolase activity (Lass et al., 2006). In addition, PKA, together with phosphorylated PLIN1, promote HSL translocation from the cytosol to the lipid droplet surface, inducing an acute activation of TG hydrolysis in conjunction with ATGL and MGL (Strålfors et al., 1984; Arner and Langin, 2014).

It should be noted that products and derivatives liberated through lipolysis do not only serve as substrates for metabolic fuel, as it was first thought, but also influence key signaling pathways involved in energy homeostasis. For instance, it has been demonstrated that lipolysis-derived fatty acids are shuttled by fatty acid-binding proteins (FABPs) to the nucleus, where they are necessary for proper peroxisome proliferator-activated receptor (PPAR) signaling (Mottillo et al., 2012). These lipids stimulate expression of target genes through either direct (as ligands of PPAR) or indirect (as precursors for other PPAR ligands) interaction with PPARs. Accordingly, ATGL-mediated lipolysis is essential for PPARα signaling in cardiac muscle and its ablation in mice leads to impaired thermogenesis due to reduced PPARα binding to BAT-specific genes such as UCP1 (Ahmadian et al., 2011; Haemmerle et al., 2011). Furthermore, the HSL-mediated reaction provides ligands to activate central adipogenesis transcription factors, including PPARγ and/or retinoid X receptor alpha (RXRα) (Ström et al., 2009; Shen et al., 2011). Therefore, lipolysis *per se* is multifunctional (Figure 1).

## The Alternative, Non-Canonical Pathway of Lipolysis: Lipophagy

In addition to the above-mentioned canonical lipase-mediated lipolysis, autophagy-based lipophagy offers an alternative mechanism for the control of fat mobilization and breakdown in various cell types, including hepatocytes (Singh et al., 2009; Zechner and Madeo, 2009), enterocytes (Khaldoun et al., 2014), macrophages (Ouimet et al., 2011), brown adipocytes (Zhang et al., 2020) and neurons (Martinez-Lopez et al., 2016).

Under conditions of nutrient deprivation, induced by the major metabolic kinases mTORC1 (Farah et al., 2016; Martinez-Lopez et al., 2016) and AMPK (Garcia and Shaw, 2017; Seo et al., 2017), lipid droplets (LDs) and autophagic machinery (phagophore) associate to form autophagic vacuoles (autolipophagosome) and subsequently autophagy promotes lipid breakdown by releasing engulfed LDs to lysosomes for degradation (Singh et al., 2009; Zechner et al., 2017). Lysosomal acid lipases are ultimately responsible for the acid hydrolysis of the LD-stored neutral lipids and subsequent release of free fatty acids (Dubland and Francis, 2015). Ever since its discovery in 2009 in hepatocytes, lipophagy has emerged as a significant component of lipid metabolism and serves a critical role in maintaining overall lipid homeostasis in physiological status (Singh et al., 2009; Zechner and Madeo, 2009). There are many excellent reviews of this topic (Schulze et al., 2017; Zechner et al., 2017; Zhang et al., 2018; Filali-Mouncef et al., 2021).

As autophagic engulfment of cytosolic cargo is size-restricted, only tiny or smaller lipid droplets can be directly targeted by lipophagy, whereas larger lipid droplets seem to be preferentially broken down into smaller ones *via* lipolysis (Schott et al., 2019). Therefore, lipolysis and lipophagy are sequential pathways, these two paralleled and distinct pathways function in a coordinated manner contributing to the regulation of the lipid turnover and the lipid metabolism homeostasis (Zechner et al., 2017; Filali-Mouncef et al., 2021).

Defective lipophagy has been linked to numerous metabolic disorders such as fatty liver (Carotti et al., 2020), obesity (Yang L. et al., 2010) and atherosclerosis (Ouimet et al., 2011), and the age-dependent decrease in autophagy (Cuervo, 2008) could underline the basis for the metabolic syndrome of aging (Singh and Cuervo, 2012). Therefore, beyond lipid mobilization, lipophagy can be considered as a defensive mechanism against ectopic lipid accumulation (Zechner and Madeo, 2009). Activation of lipophagy is a viable therapeutic target to prevent or treat such diseases where canonical lipolysis stimulation is not achievable. Generally, nutrient abundance inhibits lipophagy, while energy deprivation promotes it. Consistently, HFD feeding suppresses autophagy in mouse liver (Yang L. et al., 2010). Fatty acids are also known to regulate lipophagy. Acute stimulation with oleic acid activates lipophagy in hepatocytes (Singh et al., 2009). This activation is considered as a mechanism to buffer excess lipid influx into the cell. Nevertheless, despite considerable progress has been made in understanding lipophagy, specific molecular processes of lipophagy remain largely unknown. In addition, understanding the molecular brakes of lipophagy may provide a new approach to increase autophagic function to prevent metabolic disorders.

## Aberrant Lipolysis in Health and Disease

Adipocytes are the cell type specialized in the regulation of lipid metabolism and energy storage for the body in the form of lipids, while cells in other tissues like skeletal muscle and liver are not suited for the safe long-term storage of lipids (Pond, 1992; Li et al., 2015). The phenomenon of chronic and excess lipid accumulation in non-adipose tissues that eventually leads to IR and inflammation in the targeted tissue is termed lipotoxicity (Engin, 2017). Lipotoxicity has emerged as a key feature underlying the development of the metabolic syndrome, which itself is a major risk factor for the development of T2D and cardiovascular disease (Ballantyne et al., 2008; Unger and Scherer, 2010). The root cause for lipid accumulation in non-adipose tissues is a prolonged metabolic imbalance between lipid release and uptake as seen in obesity. An overload of energy is usually safely stored in WAT in the form of lipids, but only until the fat storage capacity of the adipocytes is reached (Gray and Vidal-Puig, 2007). Adipocytes that reached their fat storage capacity can no longer incorporate lipids from the circulation and turn into a state of lipid spillover which leads to a release of FFAs into the circulation, increasing the already high plasma lipid levels caused by chronic energy overload (Katsanos, 2018). High plasma levels of FFA lead to systemic IR *via* at least partially negating the anti-lipolytic effect of insulin on adipose tissue (Carlson et al., 2007). Consequentially, an ever-increasing level of FFA in the plasma, supported by the now uncontrolled lipolysis, eventually leads to ectopic lipid buildup and its tissue specific pathologic consequences (Schaffer, 2003).

The pathologic consequences of ectopic lipids are not primarily based on the buildup of intracellular TGs or FFAs and their hydrophobic effects on the cellular organelles. So called bioactive lipids, namely DGs and ceramides that derive from the intracellular FFA and TAG pool have been causally linked to the development of lipid-induced IR. DGs are lipid species that occur as intermediates during the esterification of FAs to TGs. Hepatic accumulation of DGs, especially sn-1,2 DG, activates PKC (protein kinase C) isoform ε, which can phosphorylate and inhibit the activity of the insulin receptor, reducing the responsiveness of the liver to insulin (Lyu et al., 2020). A similar mechanism was also proposed in DG-induced IR in skeletal muscle (Timmers et al., 2008).

Ceramides, key molecules for the biosynthesis of sphingolipids, are mostly synthesized from the saturated fatty acid thioester palmitoyl CoA and are heavily implicated in the development of IR (Holland et al., 2007; Young et al., 2012). Ceramides have emerged as potent signaling factors that are able to stimulate a number of kinases and phosphatases, most importantly PKC isoform ζ and PP2A (protein phosphatase 2A), which lead to an impairment of the insulin-dependent membrane recruitment of protein kinase B (PKB, also named as AKT) (Ruvolo, 2003; Stratford et al., 2004).

An uncontrolled basal lipolytic rate in adipocytes appears to be a key influencing factor in the development of IR and lipotoxicity. Unrestrained lipolysis in humans with mutations in PLIN1 leads to severe IR (Gandotra et al., 2011). The severity of IR varies depending on which fat depot becomes exceedingly enlarged upon prolonged metabolic imbalance. Especially, the enlargement of visceral WAT is closely correlated with IR since it exhibits a high basal lipolytic rate and secreted FFAs directly go to the liver *via* portal circulation, interfering with the liver’s regulatory role in glucose metabolism (Koska et al., 2008). In contrast, enlargement of subcutaneous fat seems to be rather metabolically protective (Suárez-Cuenca et al., 2021).

The importance of aberrant adipocyte lipolysis is highlighted by the observation that partial inhibition of adipocyte lipolysis can improve insulin sensitivity and glucose metabolism, as seen with genetic and pharmacological inhibition of HSL or in adipocyte-specific ATGL-KO mice (Girousse et al., 2013; Schoiswohl et al., 2015). Additionally, having a higher number of smaller adipocytes appears to be protective against the development of IR and the metabolic syndrome (Roberts et al., 2009). The less adipocytes one has, the more of these adipocytes are of a very large and hypertrophic phenotype that already reached their capacity for lipid storage and exhibit elevated rates of basal lipolysis (Wueest et al., 2009). Indeed, people with obesity who are still metabolically healthy have more numerous and smaller adipocytes compared to people with obesity with metabolic syndrome (Weyer et al., 2000; Cotillard et al., 2014). Of note, both decreased and increased adipocyte lipolysis have been demonstrated to improve metabolic health (Ahmadian et al., 2009; Jaworski et al., 2009). These contradictory results clearly depend on specific contexts. For patients with T2D, targeting the lipolysis to reduce the circulating FFA levels might be an effective way, while for the prevention of obesity development, boosting lipolysis might be a conceivable strategy.

Uncontrolled lipolysis is also an important factor in the cachexic process. Cancer-associated cachexia is a state of progressive metabolic disturbance that leads to muscle and adipose tissue wasting in affected patients (Tisdale, 2009). The main mechanism responsible for the marked loss of fat mass is related to strongly elevated lipolysis. Cachexic cancer patients exhibit a twofold increase in basal lipolytic activity as compared to weight-stable cancer patients and weight-loss patients due to reasons other than cachexic cancer. Mature adipocytes derived from these cachexic cancer patients expressed elevated mRNA and protein levels of HSL and showed a 2–3-fold stronger lipolytic response to catecholamines and natriuretic peptide as compared to adipocytes derived from weight stable and weight loss controls (Agustsson et al., 2007). In line with these findings, subcutaneous fat of cachexic cancer patients was additionally found to express elevated protein levels of the pro-lipolytic β-1 adrenergic receptor (Cao, 2010). These findings further highlight the importance of uncontrolled lipolysis as the driving force behind the undesired and detrimental loss of fat mass in cancer-associated cachexia.

Although inhibition of uncontrolled lipolysis seems to be promising to improve insulin sensitivity in obesity and prevent fat loss in cancer cachexia, functional basal lipolysis is essential for maintaining adipogenesis, adipocyte function as well as human health. In fact, as already mentioned, lipase-mediated lipolysis generates endogenous ligands for nuclear receptors such as PPARγ and RXRα, which are required for transcriptional regulation of adipogenesis and maintenance of adipocyte function (Itoh et al., 2008; Ström et al., 2009). As such, HSL deficiency in human patients results in impaired adipocyte development and high plasma lipid levels which eventually leads to ectopic lipid accumulation and IR (Albert et al., 2014). These metabolic phenotypes highlight the physiological significance of functional basal lipolysis in adipocyte function and systemic metabolic homeostasis and underscore the severe metabolic consequences of non-functional lipolysis. Therefore, a dynamic and delicate regulation of lipolysis is crucial for a healthy metabolism.

## Extracellular Hormonal and Autocrine/Paracrine Brakes of Adipocyte Lipolysis

**FIGURE 2:**
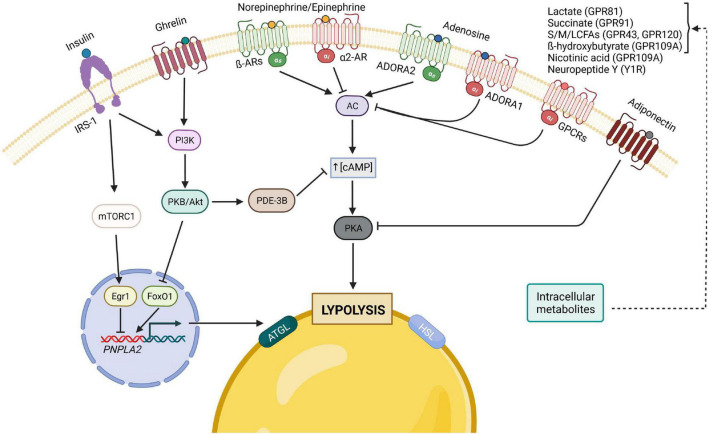
Principal hormonal, autocrine/paracrine modulators of adipocyte lipolysis. The binding of insulin to its receptor, IRS-1, leads to the activation of both PI3K/Akt and mTORC1 pathways, which result in the inhibition of lipolysis, either through the decrease of cAMP levels by PDE-3B (PI3K pathway) or by inhibiting the transcription of ATGL. The gut hormone ghrelin is also considered anti-lipolytic since it stimulates the PI3K pathway as well. Furthermore, lipolysis can also be inhibited by adiponectin, which inhibits PKA, through the reduction of its regulatory subunit of RIIα. In contrast, many of lipolysis’ regulators act on the pro-lipolytic PKA pathway, which is typically activated by catecholamines and can be inhibited by different factors upon binding to their respective Gi-coupled receptors. Of note, catecholamines and adenosine can both have pro- and anti-lipolytic effects depending on which receptor is predominantly expressed. AC, adenylyl cyclase; AR, adrenergic receptor; ATGL, adipose triglyceride lipase; cAMP, cyclic adenosine monophosphate; Egr1, Early growth response protein 1; FoxO1, forkhead box protein O1; GPCRs, G-protein-coupled receptors; HSL, hormone-sensitive lipase; IRS, insulin receptor substrate; mTORC1, mammalian target of rapamycin complex 1; PDE-3B, phosphodiesterase 3B; PI3K, phosphoinositide 3-kinase; PKA, protein kinase A; PKB, protein kinase B; NE/E, norepinephrine/epinephrine; ADORA1, adenosine receptor A1; ADORA2, adenosine receptor A2; S/M/LCFA, short-/medium-/long-chain fatty acid.

The subtle balance between fat storage and mobilization is important for the whole-body energy homeostasis; its disruption has been implicated as contributing to the development of IR, T2D and cancer cachexia. As a result, adipocyte lipolysis requires delicate regulations at the physiological level by hormones that have opposing effects as well as by autocrine/paracrine feedback mechanisms to provide a balance between energy storage and mobilization (Figure 2).

### Insulin

Insulin is the most potent antilipolytic hormone among the major signaling regulators of lipolysis such as catecholamines and natriuretic peptides (Carmen and Victor, 2006; Nielsen et al., 2014). Insulin is an anabolic hormone in mammals, produced by beta cells of the pancreatic islets when sensing high blood sugar levels, a signal of energy surplus (Baumgard et al., 2016). One of its key physiological functions is to restrain lipolysis, suppress hepatic glucose production and promote glucose uptake for *de novo* lipogenesis in the postprandial state. The failure of insulin to suppress lipolysis in adipocytes has been long considered as a crucial risk factor for the development of IR and diabetes mellitus.

Although understanding the mechanisms by which insulin suppresses adipocyte lipolysis is critical to develop potential therapeutic strategies to mitigate IR and diabetes mellitus, the detailed mechanisms remain controversial and incomplete.

The prevailing model for explaining the potent antilipolytic effect of insulin is that insulin signaling activates phosphodiesterase 3b (PDE3B) through phosphorylation at Ser273 *via* the PKB, a key downstream target for signals that activate phosphoinositide 3-kinase (PI3K). Activated PDE3B hydrolyzes cAMP to 5′-AMP thereby reducing the cAMP levels and, therefore, PKA activity (McTernan et al., 2002; Omar et al., 2011). However, several studies have challenged this and suggested that whilst PDE3B is essential under certain conditions, its function alone cannot fully explain the antilipolytic action. Instead, under low, physiological levels of adrenergic stimulation, insulin acts *via* an AKT-independent, but PI3K-dependent pathway, to inhibit PKA activity locally on the lipid droplet, possibly due to activation of serine/threonine protein phosphatases (Choi et al., 2010). Additionally, insulin may inhibit lipolysis by decreasing transcription of ATGL *via* the mTORC1-mediated pathway (mTORC1-Egr1-ATGL) or through FoxO1 (Chakrabarti and Kandror, 2009). Moreover, beyond promoting phosphodiesterase-mediated cAMP degradation, the mechanism underlying the insulin-induced reduction in adipocyte cAMP levels is more complex and involves insulin-stimulated release of lactate from adipocytes to inhibit cAMP formation *via* GPR81 (Ahmed et al., 2010). Gi-coupled receptor GPR81 is specially expressed in adipocytes and serves as a lactate receptor. Gi signaling mainly inhibits the cAMP dependent pathway by inhibiting adenylate cyclase activity, decreasing the production of cAMP from ATP.

Within this general concept, similarly, ligands of Gi-coupled receptors, such as succinate, short-chain fatty acids, medium- and long-chain fatty acids (LCFAs), ketone body β-hydroxybutyrate (β-OHB), nicotinic acid and neuropeptide Y, can also conduct anti-lipolytic effects *via* binding to their respective Gi-coupled receptors GPR91, GPR43, GPR120, GPR109A and Y1R to inhibit cAMP formation (Tunaru et al., 2003; Taggart et al., 2005; Ge et al., 2008; Regard et al., 2008; Vogel et al., 2020). These signaling ligands are important autocrine/paracrine regulators of lipolysis and are able to fine-tune the lipolytic process to the demands of different metabolic states.

### Ghrelin

The hunger hormone ghrelin is classically known as a central appetite-stimulating hormone. It is predominantly produced in the stomach and released as an anticipatory signal prior to a meal, while it decreases immediately after. Beyond its physiological role in meal initiation, previous studies revealed that both acylated and unacylated ghrelin directly attenuate adrenoreceptor-stimulated lipolysis in WAT through activation of phosphoinositide 3-kinase γ and PDE3B, likely as a mechanism to decrease fatty acid mobilization prior to the consumption of meals (Zalatan et al., 2001; Muccioli et al., 2004; Baragli et al., 2011). Of note, specie differences in the ghrelin regulation of lipolysis have been observed. In humans, ghrelin infusion acutely induces lipolysis (Vestergaard et al., 2008, 2011). It should be noted that ghrelin infusion fails to mimic the secretory pattern of ghrelin produced endogenously (Vestergaard et al., 2008). Therefore, the physiological significance of the direct metabolic effects of endogenous ghrelin remains unclear.

### Adiponectin

Adiponectin, also known as Acrp30, AdipoQ, apM1 or GBP28, is an adipocyte-derived hormone playing a crucial role in protecting against obesity-linked metabolic diseases including IR/diabetes and atherosclerosis (Yamauchi and Kadowaki, 2013). In addition to exert insulin-sensitizing effects, adiponectin has been shown to inhibit lipolysis by suppressing PKA–mediated HSL activation. In addition, adiponectin reduces the amount of PKA RIIα, the regulatory subunit of PKA, by impairing its protein stability. Overexpression of RIIα abolishes the inhibitory effects of adiponectin on lipolysis (Qiao et al., 2011). Consistently, adiponectin-knockout mice and their primary adipocytes exhibit an increased lipolysis (Qiao et al., 2011). Of note, adipose tissue expression and circulating concentrations of adiponectin are decreased in both overweight and obesity, thereby implying a plausibly decreased anti-lipolytic effect on overall lipolysis. Taken together, adiponectin, on one hand potentiates insulin-mediated suppression of lipolysis, on the other inhibits lipolysis by itself, at least.

### Catecholamines as Ligands of α2-Adrenergic Receptors

Although lipolysis is the general observed outcome of catecholamines (epinephrine and norepinephrine, also called adrenaline and noradrenaline) stimulation, it is the net result of competition between two opposing pathways (pro-lipolytic and anti-lipolytic) triggered by the same signal. Adipocytes express a combination of five different adrenoreceptor isoforms: α1, α2, β1, β2, and β3. Binding of catecholamines to the β-adrenoreceptors activates AC *via* a stimulatory G-protein (Gs). The same signal bound to the α2-adrenoreceptor affects an inhibitory G-protein (Gi), which inhibits the activity of AC. As such, lipolysis is controlled through the balanced control of lipolytic β-AR and α2A-adrenergic receptor (ADRA2). In fact, catecholamines have greater affinity to α- than to β-receptors, thus the receptor abundance (α2/β-AR ratios) largely determines which signaling modules will be activated. At very low agonist concentrations, only the α2 receptor activity is observed (anti-lipolysis). As the agonist concentration is increased, β1 becomes active and initiates lipolysis. Only under much more stimulatory agonist conditions do β3 receptors become active and promote lipolysis (Hoffstedt et al., 1995). In human fat cells, where α2-AR outnumber β-AR, the preferential activation of the α2-AR leads to an inhibition of lipolysis at low catecholamine concentrations. In small mammals, owing to the much higher expression levels of β-ARs compared with ADRA2, the stimulatory action prevails. The coexistence of pro-lipolytic β-AR and anti-lipolytic α2-AR in fat cells raises questions about their physiological relevance, which remains elusive. Notably, adipocytes from obese humans have increased α2-ARs, α2/β-AR ratios, and α2-AR-mediated responses, suggesting an important role of α2/β-AR balance in regulating lipolysis and energy balance.

### Adenosine

Local autocrine/paracrine cytokines secreted from adipocytes also regulate lipolysis. Adenosine is an endogenous purine nucleoside produced intracellularly and extracellularly in adipose tissue. It is involved in many physiological processes by binding to its four different G-protein-coupled adenosine receptors (A1, A2A, A2B, and A3 subtypes) (Hasko et al., 2008). In white fat, adenosine receptor 1 (ADORA1) is predominantly expressed and attenuates lipolysis by Gi-protein coupled pathway, once activated by adenosine, which has been validated in both mice and humans (Vernon et al., 1991; Heseltine et al., 1995; Morin et al., 2001; Johansson et al., 2008; Braun et al., 2018). In fact, it has been suggested that adenosine may serve as a physiologic feedback regulator of cAMP metabolism and lipolysis in fat cells. Nevertheless, lipolysis in the WAT of ADORA1-defecient mice did not increase, possibly owing to pleiotropic functions of ADORA1 expressed in other tissues, therefore a tissue specific knockout of ADORA1 is required for *in vivo* validation (Johansson et al., 2008). In human and murine brown fat, ADORA2A is the most abundantly expressed adenosine receptor, which is coupled with a Gs protein. Accordingly, Gnad et al. (2014) demonstrated that adenosine exerted pro-lipolytic action and thermogenic effects by stimulating AC activity through Gs-coupled ADORA2A signaling in brown fat. Therefore, similar to catecholamines, the pro- or anti-lipolytic action of adenosine largely depends on the balance of ADORA1 and ADORA2A expression.

## Intracellular Brakes on Adipocyte Lipolysis

**FIGURE 3:**
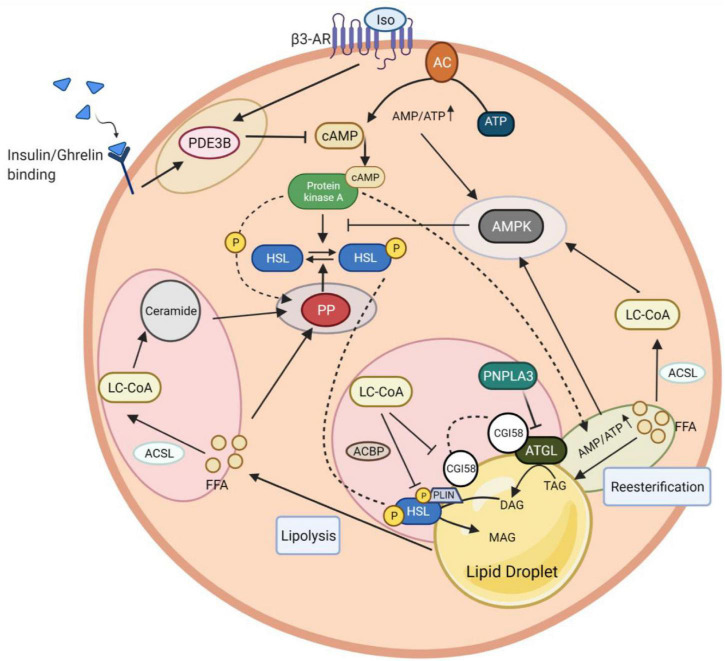
Intracellular networks regulate adipocyte lipolysis brake. The figure illustrates five feedback mechanism loops of lipolysis marked with different colors. Insulin and isoproterenol regulate lipolysis inhibition through PDE3B-mediated cAMP degradation (yellow circle). The fine-tuning of cAMP activation involves AMPK to induce a negative feedback loop in lipolysis. AMPK stimulation is coordinated *via* interaction with LC-CoA, which results from ACSL-mediated FFA conversion (light blue circle). Besides LC-CoA, cAMP production and degradation together with re-esterification, participate in this AMPK induction due to an increase of the AMP/ATP ratio yielded during these processes. PKA can potentially activate a re-eseterification enzyme called DGAT (green circle). Elevated AMPK promotes a negative feedback loop of lipolysis by inhibiting PKA-mediated HSL phosphorylation. In contrast to PKA, HSL phosphorylation can be reversed by PP, whose activation highly relies on the presence of FFA and PKA to induce a feedback mechanism (purple circle). The intermediary lipid metabolite, LC-CoA, can enter the ceramide biosynthesis pathway, followed by lipolysis attenuation *via* PP2A activation. The dynamic regulation of endogenous LC-CoA is also shown through its impact on protein lipases (HSL, ATGL) depletion. LC-CoA acts as a non-competitive inhibitor, where it binds to CGI58 and interacts with PLIN. The LC-CoA-CGI58-PLIN complex results in the deactivation of ATGL. LC-CoA also impairs HSL function enhanced by ACBP. Similar to LC-CoA, PNPLA3 serves as a competitive inhibitor of CGI58 to attenuate the ATGL catalytic activity (pink circle). PDE3B, phosphodiesterase 3B; cAMP, cyclic adenosine monophosphate; PKA, protein kinase A; AMPK, AMP-activated protein kinase; DGAT, diglyceride acyltransferase; LC-CoA, long-chain fatty acyl–CoA esters; ACSL, long-chain-fatty-acid—CoA ligase; FFA, free fatty acid; AMP/ATP, adenosine monophosphate/adenosine triphosphate ratio; HSL, hormone-sensitive lipase; PP2A, protein phosphatase 2A; ATGL, adipose triglyceride lipase; CGI-58, comparative gene identification 58; PLIN1, perilipin 1; ACBP, acyl-CoA-binding protein; PNPLA3, patatin-like phospholipase domain-containing protein 3.

In addition to the regulation by extracellular hormonal and autocrine/paracrine signals, lipolysis is also controlled by intracellular mechanisms that involve triglyceride-fatty acid (TG-FA) cycling, intracellular energy state sensing, intermediary lipid metabolites, as well as protein-protein interactions mediated modification of key components of the lipolytic signaling pathways (Figure 3).

### Reesterification

Adipose tissue lipolysis is stimulated much beyond the demand for total body fat oxidation. Existing evidence suggests that even during fasting, as much as 30–40% of lipolyzed FFA are reesterified back into TGs in rat adipose tissue and 40% in healthy humans, providing a mechanism to limit FFA release into the circulation (Reshef et al., 2003). As such, adipose tissue serves as a main source as well as a sink for fatty acid metabolism. Pathophysiologically, increased fatty acid output in diabetes has been demonstrated, at least in part, resulting from reduced esterification of fatty acids in adipocytes (Guilherme et al., 2008).

Mechanisms involved in the control of TG-FA cycling are not exactly clear. TG-FA cycling is under both hormonal and substrate control (Miyoshi et al., 1988). It is believed that the primary factors determining the extent of reesterification within the adipocyte is the intracellular FFAs buffering *via* FABPs the ability of plasma to carry away released FFA (i.e., blood flow and adequate albumin binding sites), and the availability of glucose to produce glycerol 3-phosphate for reesterification. Moreover, the absolute rates of reesterification of FFAs are reported to increase proportionally with the rate of lipolysis (Reshef et al., 1970; Brooks et al., 1982). In any case, the molecular trigger for re-esterification remains elusive. It is reasonable to speculate that lipolytic signaling pathway may activate enzymes responsible for re-esterification such as acyl CoA:diacylglycerol acyltransferase 1 (DGAT1) in parallel with activation of lipases. In fact, it has been reported that DGAT1 can be phosphorylated by PKA-dependent pathway (Yen et al., 2008). Therefore, PKA might phosphorylate and activate DGAT1 during lipolysis to promote reesterification to control intracellular FFAs level.

The fatty acid flux initiated by adipose fat lipolysis is mostly futile (Matsuo-Kato and Fujisawa, 1975). One intriguing question would be: what is the physiological significance? It has been formulated that the futile nature of fatty acid cycling may allow for its fine-tuning in response to metabolic demands (Chitraju et al., 2017). Fatty acid cycling in excess of oxidative demands allows for a rapid response to sudden changes in energy requirements, providing sufficient amounts of FFAs during energy demanding phases, while also quickly reducing FFAs availability when energy demands decline. In another scenario, the function of FFAs re-esterification in WAT during lipolysis may serve to protect the adipocyte ER from lipid-induced ER stress (Chitraju et al., 2017).

Because fatty acid re-esterification is itself a futile cycle (6 molecules of ATP are required to activate 3 molecules of fatty acid to 3 acyl-CoAs for TG synthesis, more ATPs are required depending on the origin of glycerol 3 phosphate), futile TG-FA cycling could contribute to heat production, but evidence for this hypothesis is still lacking. Moreover, the extent to which futile TG-FA cycling contributes to whole-body thermogenesis under physiological conditions is still not defined.

### AMP-Activated Protein Kinase

Stimulation of adipocyte lipolysis through PKA activation triggers, in turn, a negative feedback mechanism involving AMP-activated protein kinase (AMPK) to match the rate of lipolysis for energy supply. AMPK, a family member of serine/threonine kinases, functions as a fuel sensor in cells and plays a key role as a master regulator of cellular energy homeostasis through phosphorylating downstream metabolic proteins and transcription factors, to increase energy production and reduce energy consumption (Daval et al., 2006). AMPK is activated in adipocytes during lipolysis. Upon activation, AMPK induces the inhibitory phosphorylation (Ser565) of HSL to impede active Ser563 phosphorylation by PKA, thereby inhibiting PKA-mediated activation of HSL and lipolysis (Garton et al., 1989; Daval et al., 2005; Kim et al., 2016). The activation of AMPK in adipocytes by lipolytic agents that increase intracellular cAMP level appears to be secondary to an increase in the AMP:ATP ratio that accompanies lipolysis, rather than the direct result of increases in cAMP levels and PKA activity (Gauthier et al., 2008). In this context, evidence suggests that AMPK is activated as a consequence of constitutively ongoing reesterification that consumes significant amounts of cellular ATP and generates AMP (Gauthier et al., 2008). More recently, a study reported that AMPK is activated by interaction with long-chain fatty acyl–CoA esters (LCFA-CoAs), which offers a direct feedback loop for lipolysis on AMPK signaling (Pinkosky et al., 2020). Taken together, adipose lipolysis is also regulated by the intracellular energy state, as sensed by AMPK, for the maintenance of cellular TG homeostasis.

### Intermediary Lipid Metabolites: Free Fatty Acids, LC-CoAs and Ceramides

Lipolysis is also controlled by intracellular, intermediary lipid metabolites. Lipolysis derived FFAs, LC-CoAs (long-chain acyl-coenzyme As) and their derivatives are regarded not only as intermediates of lipid metabolism but also as potent regulators of metabolic enzymes and various signal-transducing effectors. It is well known that FFAs exert a negative feedback effect on lipolysis of adipose tissue, although possibly metabolites of FFAs, rather than FFAs *per se*, are responsible for the observed inhibition. The inhibition of FFAs on lipolysis is associated with a reduced production of cAMP due to AC inhibition, which can be prevented by reducing intracellular FFAs level *via* extracellular albumin scavenging (Burns et al., 1975, 1978; Fain and Shepherd, 1975).

The presence of excess FFA would be expected to increase intracellular levels of fatty acyl CoA as a consequence of the reaction catalyzed by fatty acyl CoA synthetase. In fact, lipolysis derived FFAs need to be activated to metabolizable LC-CoAs *via* the long-chain acyl coenzyme A synthetase (ACSL) for further metabolization such as β-oxidation, synthesis of complex lipids, or protein acylation (Faergeman and Knudsen, 1997). Previous studies demonstrated that LC-CoAs, most notably oleoyl-CoA, inhibit HSL and ATGL activity by non-competitive inhibition (Jepson and Yeaman, 1992; Nagy et al., 2014). Mechanistically, LC-CoA acts as an endogenous allosteric regulator of lipid droplet-associated protein ABHD5/CGI58, an essential co-activator of PNPLA2/ATGL. The lipase-promoting activity of CGI58 is suppressed when bound to PLIN proteins on lipid droplets. Upon binding to CGI58, LC-CoA rapidly modulates the interaction of CGI58 with perilipin proteins, thereby preventing its binding to ATGL and the activation of ATGL (Sanders et al., 2015). Moreover, the protein-protein interaction between CGI-58 and PNPLA3 (patatin-like phospholipase domain-containing protein 3, also known as adiponutrin) is also promoted by oleoyl-CoA (Yang et al., 2019). PNPLA3 competes with PNPLA2/ATGL for binding to CGI-58 and suppresses CGI-58-dependent lipolysis. Furthermore, the inhibitory effect of LC-CoA on HSL activity is dependent on the phosphorylation state of HSL and further enhanced by interactions with acyl-CoA-binding protein (ACBP) (Hu et al., 2005). As such, it is possible that LC-CoAs *via* phosphatase/ACBP/HSL/LC-CoA or phosphatase/FABP/HSL/LC-CoA complex are involved in the inhibitory effect on HSL (Hu et al., 2005).

Acyl-CoAs can further enter the ceramide-biosynthesis pathway (Summers et al., 2019). Ceramides limit lipolysis by activating the PP2A-dependent dephosphorylation of HSL (see section “Protein Phosphatases”).

Overall, these studies demonstrate that lipolysis is highly regulated by intermediary metabolites of FAs. Physiologically, these intermediary lipid metabolites provide an elegant negative feedback mechanism promoting highly effective inhibition of adipocyte FFA release, reducing FFA concentrations, avoiding FFA-mediated lipotoxicity, and promoting the storage of inert TG.

### Phosphodiesterases

Cyclic AMP and cyclic GMP are important second messengers in the signaling pathways that mobilize fat stores. Phosphodiesterases (PDEs) are a superfamily of enzymes that hydrolyze cAMP and cGMP into AMP and GMP, respectively (Kelly and Brandon, 2009). PDE enzymes are believed to act locally to control the “pool” of cAMP that activates the cAMP-effector. Therefore, activation of PDE increases degradation of cAMP and thus attenuates PKA activity, resulting in net dephosphorylation of HSL and reduced lipolysis. PDEs constitute a large and complex superfamily that contains 11 PDE gene families (PDE1 to PDE11) (Conti and Beavo, 2007; Kelly and Brandon, 2009). Some PDE families specifically hydrolyze cAMP (e.g., PDE4, PDE7, and PDE8), while some are cGMP-specific (e.g., PDE5, PDE6, and PDE9), and others can hydrolyze both substrates (e.g., PDE1, PDE2, PDE3, PDE10, and PDE11) (Snyder et al., 2005). PDE3B and PDE4 are the main PDEs expressed in adipocytes (Larsson et al., 2016). Consistent with the hypothesis that activation of PDEs dampens lipolysis, activation of PDE3B not only mediates the anti-lipolytic effect of insulin (see section “Insulin”) and ghrelin (see section “Ghrelin”), but is also involved in intracellular calcium-induced anti-lipolysis in human adipocytes (Xue et al., 2001). However, it is not clear how intracellular calcium leads to the activation of PDE3B. It is known that in addition to insulin, lipolytic hormones and other agents that increase cAMP levels, including isoproterenol, induce rapid activation of PDE3B (Pawlson et al., 1974; Makino and Kono, 1980). In rodents, lipolytic action of parathyroid hormone (PTH) is limited by simultaneous activation of PDE4 (Larsson et al., 2016). Thus, stimulation with isoproterenol results in both increased cAMP synthesis and increased cAMP degradation. Consistent with a feedback regulatory mechanism, this is believed to contribute to fine-tuning of cAMP levels and PKA activity and thereby control of lipolysis. Regarding dephosphorylation and deactivation of PDE3B, PP2A was shown to act as a PDE3B phosphatase (Resjö et al., 1999).

### Protein Phosphatases

Strictly speaking, cAMP degradation and PKA inactivation do not *per se* terminate the lipolytic cascade. Lipolysis cessation ultimately depends on the dephosphorylation of PKA targets such as HSL and perilipin by protein phosphatases. PP-mediated dephosphorylation of phosphoproteins counteracts protein kinase activity to reach a delicate balance between phosphorylated and dephosphorylated states of proteins, thus ensuring accurate signal transduction in cells (Hunter, 1995). Although protein phosphatases receive less attention than protein kinases, recent findings demonstrated that protein phosphatases are equally critical in establishing and controlling the levels of protein phosphorylation in cells, thus participating in the regulation of many physiological processes (Bononi et al., 2011). In eukaryotes, phosphatases comprise two major super families: the serine/threonine phosphatases (PSPs) and the tyrosine phosphatases (PTPs) (Shi, 2009). While the majority of protein kinases (∼400) specifically phosphorylate serine/threonine amino acids, only ∼30 protein phosphatases are serine/threonine specific. Of those PSPs, protein phosphatase 1 (PP1) and 2A (PP2A) are two crucial serine/threonine phosphatases that account for the majority of phosphatase activity in eukaryotes, through their conserved catalytic subunit (PP1c/PP2Ac) and various regulatory subunits, which determine the spatial compartmentation and substrate specificity.

Adipocyte homogenates have been shown to contain approximately equal activities of PP1 and PP2A, lower levels of PP2C and virtually no PP2B activity (Wood et al., 1993). In adipocytes, it has been demonstrated that insulin activates PP1, which dephosphorylates HSL and perilipins that insulin exerts its anti-lipolytic effects, at least in part, by activating phosphatase and independent of its ability to lower cAMP level (Olsson and Belfrage, 1987; Stralfors and Honnor, 1989; Clifford et al., 1998). PP1 also constitutes the predominant perilipin phosphatase in adipocytes (Clifford et al., 1998). However, definitive proof remains elusive and likely requires a cell model lacking PP1 activity. In fact, PP1, PP2A and PP2C all have a significant activity against HSL (Wood et al., 1993). Clearly, additional research is needed to further pinpoint the specific phosphatase responsible for the dephosphorylation of HSL and perilipin in different physiological stimulations. Multiple studies have suggested that PP2A can be activated by the cAMP-PKA pathway *via* phosphorylation of the regulatory subunit. It seems likely that PKA-PP2A-dependent dephosphorylation may contribute to a negative feedback loop that counterbalances PKA-dependent phosphorylation (Ahn et al., 2007; Ranieri et al., 2018). The functional importance of this rapid futile cycle of phosphorylation-dephosphorylation presumably allow for a more rapid turn-off mechanism for kinase inhibition or for a reduction of noise and an enhancement of the downstream signaling response specificity (Gelens and Saurin, 2018). Hence, one can imagine a similar role for protein phosphatases in the regulation of lipolysis in adipocytes. Consistently, Kinney et al. (2010) reported that overexpression of the PP2A regulatory subunit B’ (B56α) significantly decreased HSL Ser660 phosphorylation in cultured 3T3-L1CARΔ1 adipocytes, while the knockdown of B56α enhanced hormone-stimulated HSL activation and lipolysis. This results indicate that elevated B56α/PP2A levels inhibit HSL activity and lipolysis, a possible mechanism underlying impaired adipose lipolysis in a high-fat diet-induced obese (DIO) mouse model (Kinney et al., 2010). Additionally, phospholipase C-related catalytically inactive protein (PRIP), which can bind to both PP1 and PP2A, promotes the translocation of phosphatases to the surface of lipid droplets upon adrenergic stimulation, and the subsequent dephosphorylation of HSL and perilipin, leading to inhibition of PKA-mediated lipolysis (Okumura et al., 2014). Of note, intermediary lipid metabolites such as FFAs and/or ceramides have also been reported to activate cellular phosphatase activity (Dobrowsky et al., 1993; Chalfant et al., 1999; Galbo et al., 2011). Therefore, a diversity of ways to activate phosphatase occur during PKA-mediated lipolysis.

Taken together, protein phosphatase-mediated dephosphorylation of lipolytic machinery is the most crucial and ultimate step for terminating lipolysis. Multiple levels of regulation, including signal transduction as well as intermediary lipid metabolites, converge on regulating phosphatase activity to finely tune the lipolytic response.

### Protein–Protein Interaction Based Molecular Brakes on Adipocyte Lipolysis

**FIGURE 4:**
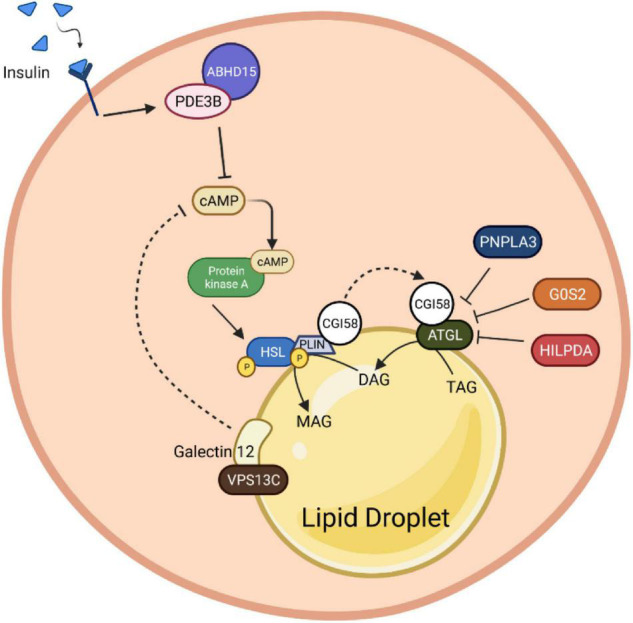
Protein-protein interaction roles in adipocyte lipolysis. Insulin binding induces stabilization of PDE3B with ABHD15. This molecular complex promotes cAMP degradation, which consequently inhibits the HSL phosphorylation upon PKA inactivation. Besides HSL, PLIN1 is also directly regulated by the PKA. The stimulation of PLIN1 mediates CGI58 release from the PLIN1 and HSL complex to bind with ATGL and induce its catalytic activity. Nevertheless, the complex of CGI58 and HSL can be suppressed by the presence of binding inhibitors, G0S2, HILPDA and PNPLA3. Presumably, Galectin12, a protein that localizes on lipid droplets, and its coactivator, VPSC13, can alter the cAMP production by inhibiting the PDE recruitment function, which in the end contributes to lipolysis abolition. PDE3B, phosphodiesterase 3B; cAMP, cyclic adenosine monophosphate; AMPK, AMP-activated protein kinase; HSL, hormone-sensitive lipase; PLIN1, perilipin 1; PKA, protein kinase A; CGI-58, comparative gene identification 58; ATGL, adipose triglyceride lipase; G0S2, G0/S2 switch protein 2; HILPDA, hypoxia-induced lipid droplet-associated protein; PNPLA3, patatin-like phospholipase domain-containing protein 3; VPSC13, vacuolar protein sorting 13C.

In addition to the above-mentioned brake nodes, phosphodiesterases, lipolytic enzymes as well as lipid droplet scaffolding proteins themselves are targets of a plethora of proteinaceous interaction partners to form large macro-molecular complexes (signalosomes) regulating their endogenous activity, thus adding another level of complexity and feedback control toward this system (Figure 4).

#### ABHD15- PDE3B-cAMP

The α/β-hydrolase domain containing protein 15 (ABHD15), initially identified as a 47 KDa AKT substrate, has been shown to form macromolecular complexes with PDE3B and regulates PDE3B protein amount in adipocytes (Onuma et al., 2005; Ahmad et al., 2007). Recent studies have shown that stabilization of PDE3B, resulting from ABHD15 binding, is necessary for insulin-stimulated suppression of lipolysis in WAT (Xia et al., 2018; Stöckli et al., 2019). Upon loss of ABHD15, insulin fails to activate PDE3B-mediated cAMP degradation to inhibit PKA activity and reduce phosphorylation of PLIN1 and HSL, leading to unsuppressed lipolysis. However, the precise molecular mechanism underling the interaction between ABHD15 and PDE3B remains elusive.

#### G0S2-CGI58-ATGL

As mentioned previously, CGI58/ABHD5 is a lipid droplet-associated ATGL activator, which interacts with PLIN1 to regulate basal and stimulated lipolysis. Briefly, the ATGL-activating function of CGI58 is repressed by its binding to PLIN1, a lipid droplet scaffold, under basal conditions. Upon activation by PKA-mediated phosphorylation of PLIN1 and CGI58, CGI58 is released from PLIN1 and LDs, which then recruits ATGL to LDs and increases its catalytic activity. Therefore, lipolysis involves interactions between ATGL, CGI58, and PLINs; CGI58 is a key molecule for lipolysis activation in adipocytes, even though the exact mechanism by which CGI58 increases ATGL activity is not completely understood. As such, factors sequestering CGI58 from ATGL would be anti-lipolytic. Accordingly, G0S2 inhibits ATGL activity and decreases lipolysis in rodents and humans with a dose-dependent, non-competitive manner even in the presence of the coactivator CGI58 (Yang X. et al., 2010; Schweiger et al., 2012). Mutagenesis analysis revealed that the central hydrophobic domain of G0S2 is required for interaction and inhibition of ATGL, as well as the N-terminal patatin-like region (up to Leu254) of ATGL (Yang X. et al., 2010; Cornaciu et al., 2011). Changes in G0S2 levels are observed throughout the feeding-fasting-refeeding cycle, suggesting that G0S2-mediated modification of lipolysis is required for rapid metabolic adaptations, possibly by interacting with CGI58 and ATGL (Zhang et al., 2014).

Beyond its well-recognized antilipolytic function, G0S2 also harbors lysophosphatidyl-acyltransferase activity and plays a critical role in promoting hepatic triglyceride synthesis (Zhang et al., 2019). This dual function, acting as both a lipid-synthesizing enzyme and an ATGL inhibitor, enables G0S2 as a master regulator of lipid storage and mobilization balance to control the intracellular levels of fatty acids.

#### HILPDA-CGI58-ATGL

Similar to G0S2, hypoxia-induced lipid droplet-associated protein (HILPDA), also known as hypoxia-induced gene-2 (HIG2), inhibits ATGL activity through direct physical interaction as well, even though with a lower inhibitory potency than G0S2 (Zhang et al., 2017; Padmanabha Das et al., 2018). As HILPDA shares a lot of functional and structural resemblance with G0S2, the reasons for being equipped with two similar, endogenous ATGL inhibitors are unclear. Hypothesis proposes that HILPDA and G0S2 may regulate distinct subpopulations of lipid droplets rather than functionally redundant has been formulated (de la Rosa Rodriguez and Kersten, 2020). Notably, HILPDA preferably accumulates in lipid droplets undergoing either shrinking or expansion. Interestingly, independently of its effects on ATGL, HILPDA also stimulates DGAT1-mediated triglyceride synthesis and promotes lipid synthesis in hepatocytes and adipocytes (de la Rosa Rodriguez et al., 2021). As adrenergic stimulation as well as fatty acids themselves increase HILPDA levels, it is of interest to test whether HILPDA regulates fatty acid re-esterification. Possibly due to mutual functional compensation, the effect of G0S2 or HILPDA deficiency in adipose tissue was relatively modest if any. Overall, HILPDA is a novel regulatory signal with dual function that reduces the intracellular concentration of free fatty acids by either inhibiting the first step in triglyceride breakdown or activating the last step in triglyceride synthesis (de la Rosa Rodriguez and Kersten, 2020).

#### Adiponutrin/PNPLA3-CGI58-ATGL

Conceptually similar to the regulation of ATGL activity by G0S2 and HILPDA (see above), adiponutrin (also named as PNPLA3), which is a close paralog of ATGL, has been demonstrated to compete with ATGL for binding to CGI58 and, in turn, effectively sequestering CGI58 from activating ATGL, thereby decreasing lipolysis (Yang et al., 2019). Genetic variations in the human PNPLA3 gene [i.e., the rs738409 I148M allele (PNPLA3I148M)] have been strongly associated with fatty liver disease (Romeo et al., 2008). How the variant predisposes individuals to non-alcoholic fatty liver disease (NAFLD) remains elusive. Surprisingly, PNPLA3-null mice exhibited no detectable phenotype in lipid and energy metabolism, thus suggesting that PNPLA3I148M is not a severe loss-of-function variant (Chen et al., 2010; Basantani et al., 2011). It turns out that PNPLA3I148M suppresses the interaction of CGI58 with ATGL even more effectively and reduces CGI58-dependent lipolysis to a greater degree. Meanwhile, it promotes TAG accumulation, providing a mechanism by which the PNPLA3I148M variant confers susceptibility to fatty liver disease. However, the details of how exactly this might occur require further investigation. Notably, overexpression of the wild-type protein in transgenic mice does not lead to fatty liver, suggesting unknown potential mechanism that could be modified by the PNPLA3–CGI58 interaction. Interestingly, the interaction of PNPLA3 and CGI58 can be increased by endogenous intermediary lipid metabolites such as long-chain acyl-CoAs and synthetic CGI58 ligands (Yang et al., 2019). Nevertheless, further studies are needed to fully assess the dynamic role of PNPLA3 in lipolysis regulation, acyl-CoA metabolism and its physiological relevance.

#### Galectin12-VPS13C

The surface of perilipin-coated lipid droplets has emerged as a central site of lipolysis regulation. A common function of many proteins that bind to lipid droplets, including perilipins, is to serve as gatekeepers to modulate lipolysis by controlling the access of lipases and their co-factors to substrate lipids stored within lipid droplets. Galectin12 is an endogenous lectin preferentially expressed in adipose tissue and located on lipid droplets in adipocytes (Yang et al., 2011). It is not yet clear how galectin12 could localize to a subset of lipid droplets. Notably, galectin12 deficiency in mice results in enhanced lipolysis, reduced adiposity, and ameliorated IR (Yang et al., 2011). Mechanistically, loss of galectin12 increases the efficiency of cAMP signaling at the lipid droplet, perhaps by altering the recruitment or function of a phosphodiesterase, and consequentially promoting PKA-dependent recruitment of lipases to lipid droplets. Nevertheless, the precise mechanism of lipolysis control by galectin12 is not clear. The critical step of understanding how galectin12 participates in lipolysis will be to identify the factors at the droplets that directly interact with galectin12. Notably, vacuolar protein sorting 13C (VPS13C), a novel lipid droplet protein, was identified as a major galectin12-binding protein that is required for galectin12 protein stability (Yang et al., 2016). Consistently, knockdown of VPS13C leaded to galectin12 degradation primarily through the lysosomal pathway (Yang et al., 2016). A more recent study revealed that VPS13C was abundantly expressed in mouse BAT (Ramseyer et al., 2018). Similarly, deletion of VPS13C in brown and white adipocytes augments basal and β-adrenergic-induced lipolysis, likely due to enhanced ATGL trafficking to lipid droplets (Ramseyer et al., 2018). It will be of interest to explore whether the impact of VPS13C loss on lipolysis depends on galectin12 or *vice versa*. Ultimately, a comprehensive understanding of the interactome of galectin12 and VPS13C on lipid droplets and identification of the components of lipid droplets proteome that harbor pro- and/or anti-lipolytic effects are important future research directions. Regardless, these results indicate that galectin12 and VPS13C functions are important for suppression of lipolysis.

## Roles of Fine-Tuned Lipolysis in Thermogenic Fat Biology

In thermogenic brown and brite adipocytes, lipolysis stimulation is a canonical way for the activation of non-shivering thermogenesis. FFAs released by lipolysis are direct activators of uncoupling protein 1-dependent thermogenesis. In other words, UCP1 activity depends on the cellular availability of FFAs. Thus, pro- and anti-lipolytic mediators are *bona fide* modulators of thermogenesis in brown and brite adipocytes (Braun et al., 2018).

Hitherto, thermogenesis had always been correlated with the presence of BAT local lipolysis. It has been reported, however, that lipolysis in white adipocytes permits maximal stimulation of energy expenditure, as β3-ARs must be present in both white and brown adipocytes in order for β3-AR agonist (CL-316,243) to maximally increase thermogenesis, while lack of β3-ARs in white adipocytes significantly limits the stimulatory effect (Grujic et al., 1997). The mechanism by which β3-ARs in white adipocytes permit maximal stimulation of brown fat thermogenesis is unknown but might be related to their roles in mobilizing FFAs. When the FFA concentration reaches a sufficient level in the blood, FFAs can be used as activators and/or fuel for energy expenditure in brown adipocytes (Grujic et al., 1997). Consistent with this scenario, more recently, when Schreiber et al. (2017) tried to knock out the ATGL and Shin et al. (2017) deleted an ATGL-activating protein CGI58 specifically in BAT, respectively, it is observed that lipolysis did not necessarily occur locally within the brown fat cells to ignite thermogenesis in both cases during cold. In line with these findings, a recent study reported that even lipid droplets in brown fat were dispensable for thermogenesis in response to cold when other fuel sources were present (Chitraju et al., 2020). By ablation of TG biosynthesis enzyme acyl CoA:DGAT specifically in brown fat, the authors found that in the absence of TG storage in brown adipocytes, other substrates, such as circulating glucose and FAs, as well as increased brown adipocyte glycogen, appear to be sufficient to compensate to fuel respiration and thermogenesis (Chitraju et al., 2020). Overall, these studies imply that with the deficiency of lipolytic machinery and even lack of energy storage in BAT, the breakdown of stored TGs in WAT is a key feature for maintaining thermogenesis. WAT lipolysis promotes FFA release, which thereafter can be deployed into BAT as activators and/or fuel for energy dissipation *via* UCP1 (Schreiber et al., 2017; Shin et al., 2017; Chitraju et al., 2020). Aside from WAT lipolysis, other sources of FFAs can be derived through lipoprotein lipase (LPL)-mediated triglyceride-rich lipoprotein (TRL) degradation. This pathway liberates FFAs into the vascular lumen. In addition, a recent study demonstrates that TRL remnants can be fully incorporated into endothelial cells, followed by lysosomal acid lipase (LAL)-induced FFA release in the cells. Released FFAs will be further uptaken by brown adipocytes to induce thermogenesis (Bartelt et al., 2017; Lynes et al., 2017; Singh et al., 2018; Fischer et al., 2021).

Inhibition of intracellular lipolysis and loss of TG synthesis enzymes inside brown adipocytes substantially promotes the import of extracellular FFAs upon thermogenesis demand. However, the mechanism through which circulating FFAs produced from WAT can be imported into BAT has not been addressed yet. The possible pathway of how cold exposure induces FFA uptake in brown fat might be mediated through increasing in LPL activity as well as various transporters such as fatty acid transport protein (FATP), FABP, and the scavenger receptor CD36 (Klingenspor et al., 1996; Wu et al., 2006; Anderson et al., 2015; Shu et al., 2017). The circulating lipid chaperone called A-FABP (or known as FABP4) has been reported to facilitate FFA import from WAT into BAT (Shu et al., 2017). FABP is a carrier protein for FFAs not only derived from extracellular lipolysis but also from intracellular lipolysis (Mita et al., 2015). It was reported that FABP-FFA complex could be internalized through endocytosis (Shrestha et al., 2018; Villeneuve et al., 2018). The loss of the integral transmembrane transporter FATP1 in murine brown fat also marks attenuation in dissipating metabolic energy as heat (Wu et al., 2006).

**FIGURE 5:**
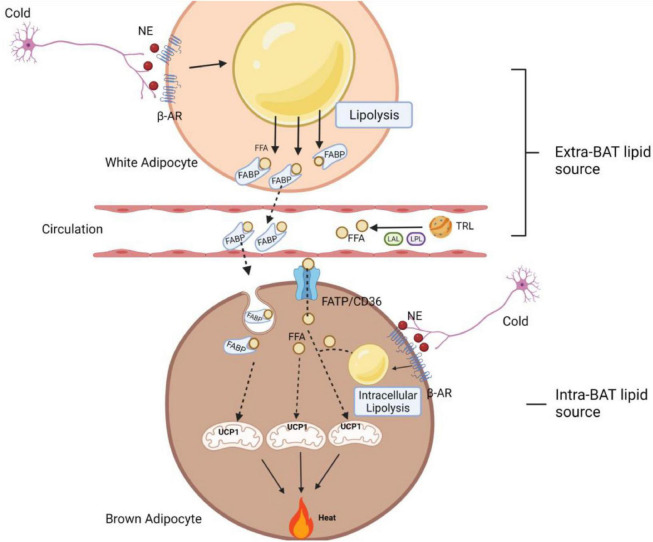
A schematic diagram of fine-tuned lipolysis in thermogenic biology. Upon cold stimulation, the non-shivering thermogenesis process in BAT can be mediated through intra- and extra-BAT FFAs source. In local lipolysis, FFAs generated from brown adipocyte lipid droplets can be used directly to activate and fuel UCP1 to produce heat. In addition, there is also a synergistic and compensatory mechanism, where white adipocyte lipolysis and TRL degradation (with the help of LPL and LAL) serve as extracellular fuels to permit a maximum thermogenesis level. These circulating FFAs are imported into BAT *via* FABP, FATP1 and CD36 transporters. Among them, FABP assists the binding of FFA released in WAT, which is later imported into brown adipocytes as a FABP-FFA complex *via* endocytosis. AR, adrenergic receptor; FFA, free fatty acid; NE, norepinephrine; FABP, fatty acid-binding protein; FATP, fatty acid transport proteins; TRL, triglyceride-rich lipoprotein; LPL, lipoprotein lipase; LAL, lysosomal acid lipase; UCP1, uncoupling protein 1.

The crosstalk between WAT and BAT in terms of the compensatory role as well as synergistic interaction for allowing maximal stimulation of adaptive thermogenesis provides evidences for a fine-tuned lipolysis in the activation of thermogenesis (Figure 5). The flexibility of UCP-1 activation through catecholamines-induced WAT lipid breakdown indicates an alternative pathway to increase energy combustion. Taken together, the concept that white adipocytes lipolysis permit maximal stimulation of energy expenditure provides significant implications for boosting energy expenditure and the treatment of human obesity.

## Conclusion and Perspective

Adipose lipolysis not only provides metabolic fuel for the whole body, but also produces products that act as signaling molecules and transcriptional modulators, which are essential for preserving adipocyte function. Aberrant lipolysis, both excessive and impaired lipolysis, is tightly linked to severe metabolic disorders such as obesity, T2D, non-alcoholic fatty liver disease and cancer cachexia. Thus, lipolysis is under tight control involving extracellular circulating hormones, local autocrine/paracrine factors, as well as delicate intracellular interplays. Intricate cooperation of these regulatory signals leads to a fine-tuning of lipolysis in adipocytes that are essential for energy homeostasis. The existence of these distinct pathways will undoubtedly influence the approach to develop therapeutic treatments that target specific components of the lipolytic signaling pathway. Our understanding of these pathways is still in its infancy and many questions remain. Generally, while stimulation of adipocyte Gs-coupled GPCRs results in cAMP-mediated activation of PKA, resulting in the phosphorylation and activation of HSL and enhanced lipolysis, activation of adipocyte Gi-coupled receptors exerts an antilipolytic effect *via* decrease in cAMP levels. This counter-regulation between G protein-coupled receptors suggests a system evolved for defending a minimal but sufficient cAMP signaling capacity while protecting against excess activation. Intracellularly, a variety of negative feedback loops targeting key components of the signaling pathways leads to further multi-layered fine-tuning of lipolysis, which exemplifies the complexity and importance of feedback controls. A common feature of these regulations is that they interfere with assembly/composition of signalosome complexes (critical nodes) *via* protein-protein interactions. However, we are just beginning to appreciate these fine-tuning. Additional complex feedback loops and the coordinated interplays with lipolytic signalosomes as well as lipolytic machinery require further investigation. A comprehensive understanding of lipolytic regulatory factors remains a challenge and will require unbiased extensive research, such as CRISPR-based genome-wide screen, to identify new components in the signaling networks and to map their connectivity.

Of note, both the stimulation and inhibition of adipocyte lipolysis have been demonstrated to improve metabolic health. As such, tailored therapeutic strategies depend on subject metabolic context. In thermogenetic brown and brite adipocytes, the canonical UCP1-mediated thermogenesis is activated and fueled by FFAs to produce heat for body temperature maintenance. Pro-lipolytic factors are potential activators of thermogenesis in brown and brite adipocytes. It must be mentioned that while the mobilization of fatty acid supply is required for thermogenesis, this actually tends to raise fatty acid metabolite levels and reduces insulin sensitivity. It is only when the increased fatty acid oxidation capacity or reesterification cycle exceeds the mobilization that consequentially fatty acid metabolite levels fall and insulin sensitivity improves. Moreover, the synergic effects of white adipocytes lipolysis with brown fat thermogenesis activation in permitting maximal stimulation of energy expenditure should be kept in mind when developing novel interventions to boost thermogenesis and treat obesity-related metabolic diseases.

## Author Contributions

YL conceived, structured, drafted, and edited the review. ML drafted the lipolysis pathway part and created Figures 1, 2. AW drafted the lipolysis in the health and disease section. DN drafted the thermogenesis part and created Figures 3–5. YW contributed to the lipophagy section. YL drafted the rest parts, with input from ZL for the brake parts. All authors read, edited, and approved the manuscript.

## Conflict of Interest

The authors declare that the research was conducted in the absence of any commercial or financial relationships that could be construed as a potential conflict of interest.

## Publisher’s Note

All claims expressed in this article are solely those of the authors and do not necessarily represent those of their affiliated organizations, or those of the publisher, the editors and the reviewers. Any product that may be evaluated in this article, or claim that may be made by its manufacturer, is not guaranteed or endorsed by the publisher.
